# Cationic Polymer-Coated Magnetic Nanoparticles with Antibacterial Properties: Synthesis and In Vitro Characterization

**DOI:** 10.3390/antibiotics10091077

**Published:** 2021-09-06

**Authors:** Anastasiia B. Shatan, Vitalii Patsula, Aneta Dydowiczová, Kristýna Gunár, Nadiia Velychkivska, Jiřina Hromádková, Eduard Petrovský, Daniel Horák

**Affiliations:** 1 Institute of Macromolecular Chemistry, Czech Academy of Sciences, Heyrovského nám. 2, 162 06 Prague 6, Czech Republic; shatan@imc.cas.cz (A.B.S.); patsula@imc.cas.cz (V.P.); dydowiczova@imc.cas.cz (A.D.); gunar@imc.cas.cz (K.G.); velychkivska@imc.cas.cz (N.V.); hromadkova@imc.cas.cz (J.H.); 2 Department of Physical and Macromolecular Chemistry, Faculty of Science, Charles University, Hlavova 8, 128 40 Prague 2, Czech Republic; 3 Institute of Geophysics, Czech Academy of Sciences, Boční II/1401, 141 31 Prague 4, Czech Republic; edp@ig.cas.cz

**Keywords:** magnetic, nanoparticles, antibacterial activity, 2-(dimethylamino)ethyl methacrylate, 2-*tert*-butylaminoethyl methacrylate

## Abstract

Uniformly sized magnetite nanoparticles (*D*_n_ = 16 nm) were prepared by a thermal decomposition of Fe(III) oleate in octadec-1-ene and stabilized by oleic acid. The particles were coated with Sipomer PAM-200 containing both phosphate and methacrylic groups available for the attachment to the iron oxide and at the same time enabling (co)polymerization of 2-(dimethylamino)ethyl methacrylate and/or 2-*tert*-butylaminoethyl methacrylate at two molar ratios. The poly[2-(dimethylamino)ethyl methacrylate] (PDMAEMA) and poly[2-(dimethylamino)ethyl methacrylate-*co*-2-*tert*-butylaminoethyl methacrylate] [P(DMAEMA-TBAEMA)] polymers and the particles were characterized by ^1^H NMR spectroscopy, size-exclusion chromatography, transmission electron microscopy, dynamic light scattering, thermogravimetric analysis, magnetometry, and ATR FTIR and atomic absorption spectroscopy. The antimicrobial effect of cationic polymer-coated magnetite nanoparticles tested on both *Escherichia coli* and *Staphylococcus aureus* bacteria was found to be time- and dose-responsive. The P(DMAEMA-TBAEMA)-coated magnetite particles possessed superior biocidal properties compared to those of P(DMAEMA)-coated one.

## 1. Introduction

The invention of antibiotics in the 1940s saved millions of lives and many infectious diseases became far less deadly. However, bacteria have currently developed resistance to antibiotics, which is now a major threat for public health [1,2,3]. Moreover, there is a need for disinfection of drinking water in remote areas using alternative approaches to chlorination, in order to eliminate pathogens responsible for waterborne diseases. There are other physical processes to disinfect the contaminated water including filtration, thermal treatment, or UV irradiation; however, these methods suffer from limitations, such as time demand, high price, waste of resources, use of various additives and agents, etc. [4]. In a search for new, cheap, and simple solutions of these problems, a significant effort has been devoted to the development of antibacterial agents based on nanoparticles [5,6,7]. In contrast to conventional antibiotics, such materials possess a range of unique physicochemical and biological properties, e.g., high surface-to-volume ratio that increases contact area with microorganisms, high stability, possibility of easy modification with various functional groups, ligands, targeting agents and other biomolecules, enabling not only disinfection, but also its monitoring and targeting [8,9,10]. Particles possessing sterilization properties are typically based on silver, copper, various metal oxides or sulfides, or carbon nanotubes [11].

Among the different types of nanoparticles, the magnetic ones especially show a great potential for various biomedical applications, such as magnetic resonance imaging, hyperthermia, drug delivery, tissue repair, and cell and tissue targeting and transfection [9]. Due to these distinct characteristics, including response to external magnetic force, it is of great interest to also explore their applicability as a carrier of antibacterial polymers. Such nanomaterials can be highly efficient biocides that would be easily manipulatable, recyclable, and reusable by using an external magnetic field. At the same time, the functional antibacterial magnetic nanoparticles based on iron oxides could overcome pathogen’s multi-drug resistibility inhibiting bacterial growth [12,13]. The antibacterial mechanism of these nanoparticles was mainly attributed to dissolved metal ions and the gene-ration of reactive oxygen species [7]. They could also electrostatically bind to the cell membrane, inducing disorder in the bacteria functions and leading to cell death [13].

Many materials and methods have been already described for iron oxide synthesis and surface modification with the aim to provide colloidal stability of the dispersions, compatibility with the living tissues, and attachment of target molecules. For example, conjugation of macromolecules to the particle surface was achieved by anchoring groups, such are aromatic vicinal diols, carboxyl, bisphosphonate, phosphate, or hydroxamate groups, capable of strong interactions with iron [14]. The strongest binding was achieved with phosphate groups present, e.g., in penta(propylene glycol) methacrylate phosphate (Sipomer PAM-200). Sipomer PAM-200 is a heterobifunctional macromonomer built of five propylene oxide units and containing a reactive methacrylic group with a double bond that allows the radical polymerization with a monomer [15,16]. Moreover, Sipomer PAM-200 is terminated with a phosphate group able to interact with the iron oxide. To confer biocidal properties to the particles and/or prevent antibiotic resistance, the particles were also modified with antimicrobial polymers that can reduce general toxicity and ensure colloidal stability [17,18,19]. Typical examples of such compounds included polymers with quaternary ammonium, pyridinium, and/or phosphonium cations [19,20,21,22,23,24]. Their bactericidal activity originated from the interaction of the cationic sites of the polymer with negatively charged membrane proteins of bacteria, which were ultimately disrupted. For example, mucoadhesive and thermoresponsive poly[2-(dimethylamino)ethyl metha-crylate] (PDMAEMA) was incorporated in antimicrobial copolymers to inhibit growth of *Staphylococcus aureus* [25,26] and *Escherichia coli* [27]. Other PDMAEMA applications included nonviral gene delivery [28], drug delivery [29], water purification [30], or protein separation [31]. In addition to PDMAEMA, another antimicrobial polymer, poly(2-*tert*-butylaminoethyl methacrylate) (PTBAEMA) that is hydrophobic was contained in various blends or capsules [32,33,34]. It displaced Ca^2+^ and/or Mg^2+^ ions from the outer bacterial membrane, which became disorganized and disrupted [33,35]. Obviously, large pendant *tert*-butylamino groups of PTBAEMA were not necessarily quaternized to become antibacterial.

The aim of this report was to anchor water-soluble antibacterial polymers prepared by free-radical polymerization of DMAEMA and TBAEMA to a magnetic carrier to make it colloidally stable, offer high surface area available for interactions with bacteria, and investigate antimicrobial activity of the particles against *E. coli* and *S. aureus*. The advantages of this new nanomaterial consist of its easy manipulation, targeting, and separation from liquid media using a magnet.

## 2. Experimental

### 2.1. Materials and Methods

Octadec-1-ene (OD), FeCl_3_·6H_2_O (98%), 2-(dimethylamino)ethyl methacrylate (DMAEMA), *tert*-butylaminoethyl methacrylate (TBAEMA), Hank’s balanced salt solution (HBSS), and Luria broth (LB) were purchased from Sigma-Aldrich (St. Louis, MO, USA). Oleic acid (OA; 95%), ethanol (99%), hexane (99%), HCl (35%), and NaOH (98.6%) were obtained from Lach-Ner (Neratovice, Czech Republic). Sipomer PAM 200 (*M*_w_ = 451 Da; acronym S) was from Rhodia (Courbevoie, France). Cellulose membrane (100 kDa) for dialysis was purchased from Spectrum Europe (Breda, Netherlands). Fe(III) oleate was prepared according to an earlier report [36]. All other chemicals were purchased from Sigma-Aldrich. Ultrapure Q-water ultrafiltered on a Milli-Q Gradient A10 system (Millipore; Molsheim, France) was used in the experiments.

Antibacterial activity of nanoparticles was evaluated using two types of cultures: Gram-positive bacteria represented by *Staphylococcus* (*S*.) *aureus* and Gram-negative bacteria *Escherichia* (*E*.) *coli*. Both bacterial strains were isolated and identified at the Institute of Immunology and Microbiology of the 1st Faculty of Medicine and General University Hospital in Prague, Czech Republic. Bacteria cultures were cultivated on Luria agar (LA) plates (Sigma-Aldrich) at 37 °C.

### 2.2. Preparation of Sipomer PAM-200-Coated Fe_3_O_4_ Nanoparticles

OA-stabilized Fe_3_O_4_ nanoparticles (MNP) were obtained by a thermal decomposition of Fe(III) oleate as reported earlier [37,38]. Briefly, Fe(III) oleate (5.76 g) was dissolved in a mixture of OD (31.61 g) and OA (4.51 g), the reaction mixture was preheated at 120 °C for 60 min under argon flow and then heated at 320 °C for 30 min. After cooling to room temperature (RT), ethanol (100 Ml) was added and the resulting particles were separated by a magnet and washed with hot ethanol (60–70 °C) three times (50 Ml each). Finally, oleic acid-stabilized Fe_3_O_4_ particles (0.2 g) and Sipomer PAM-200 (0.4 g) were dispersed in toluene (25 mL), the mixture was sonicated (20% of power) in an ice-water bath for 5 min, kept under argon atmosphere for 15 min, and stirred (900 rpm) at RT for 48 h. The resulting Sipomer PAM-200-coated Fe_3_O_4_ particles (denoted as MNP@S) were precipitated in hexane three times (100 mL each), separated by a magnet to remove residual solvents and OA, dispersed in tetrahydrofuran (THF), and stored at 4 °C.

### 2.3. Modification of MNP@S by DMAEMA-Based Polymers

DMAEMA-based homo- and copolymers were grafted on the surface of MNP@S. Briefly, DMAEMA (denoted as D) was free radically polymerized or D was copolymerized with TBAEMA (denoted as T) at 1/0, 0.75/0.25, and 0.5/0.5 molar ratios on MNP@S (50 mg) in THF (5 mL) with ACVA as an initiator (2.1 mg) at 70 °C for 18 h. The resulting poly[(2-dimethylamino)ethyl methacrylate]-, poly[2-(dimethylamino)ethyl methacrylate-*co-*2-*tert*-butylaminoethyl methacrylate] (0.75/0.25 mol/mol)-, and poly[2-(dimethylamino)ethyl methacrylate-*co-*2-*tert*-butylaminoethyl methacrylate] (0.5/0.5 mol/mol)-mo-dified MNP@S were denoted as MNP@S-D, MNP@S-D-T1, and MNP@S-D-T2, respectively. They were precipitated with hexane three times (100 mL each), separated by a magnet, redispersed in water, and dialyzed against water using a cellulose dialysis membrane (MWCO 300 kDa) for 48 h to remove excessive polymer. Finally, the particle dispersions were freeze-dried and stored. The composition and purity of PDMAEMA and its copolymers was confirmed by ^1^H NMR analysis; the following ratio was calculated: DMAEMA/TBAEMA = a_x_/m_x_: a_y_/m_y_, where a_x_ and a_y_ are the area under ^1^H NMR peak of (CH_3_)_2_ and (CH_3_)_3_, respectively, and m_x_ and m_y_ are the number of (CH_3_)_2_ and (CH_3_)_3_ protons, respectively.

### 2.4. Physicochemical Characterization

High-resolution ^1^H NMR spectra were acquired in deuterated water with a Bruker Avance III 600 spectrometer operating at 600.2 MHz and processed with the Topspin 4.0.5 software (both Bruker; Billerica, MA, USA). Measurements were as follows: 90° pulse width 10 μs, relaxation delay 10 s, spectral width 7211 Hz, acquisition time 4.54 s, and 32 scans. The integrated intensities were determined using the spectrometer integration software with an accuracy of ±1%. During the measurements, temperature was maintained at 25 ± 0.2 °C using a BVT 3000 temperature unit.

The size-exclusion chromatography (SEC) of the polymers was performed at 25 °C with a TSKgel SuperAW-L guard column (L × I.D. 4.6 mm × 3.5 cm, particle size 7 μm; Polymer Laboratories; Church Stretton, UK) and UVD 305 (Watrex; Prague, Czech Republic) and RI-101 (Shodex; Tokyo, Japan) detectors. Methanol/acetate buffer mixture (80/20 *v/v*) was used as a mobile phase at a flow rate of 0.5 mL/min. The molar mass was calculated using Clarity software (DataApex; Prague, Czech Republic) with poly-styrene as a calibration standard. The samples for both ^1^H NMR and SEC analysis were prepared by dissolution of the polymer-coated MNP in 2 M HCl; pH of the solution was adjusted to 7 and the solution was dialyzed against water using a cellulose dialysis membrane (MWCO 3.5 kDa) and purified on a Sephadex^TM^ LH-20 column (Merck; Kenilworth, NJ, USA).

Morphology, size, and size distribution of the particles were analyzed with a Tecnai G2 Spirit Twin 12 transmission electron microscope (TEM; FEI; Brno, Czech Republic) [10]. Number-average diameter (*D*_n_ = ΣN_i_*∙D*_i_/ΣN_i_), weight-average diameter (*D*_w_ = ΣN_i_*∙D*_i_^4^/ΣN_i_*∙D*_i_^3^), and dispersity (*Ð = D*_w_/*D*_n_) were calculated from at least 500 individual particles from the TEM micrographs using Atlas software (Tescan, Brno, Czech Republic).

Hydrodynamic diameter (*D*_h_), polydispersity (*PD*), and ζ-potential were obtained by dynamic light scattering (DLS) using a ZEN 3600 Zetasizer Nano instrument (Malvern Instruments; Malvern, UK) at RT and different pHs. The *D*_h_ was calculated from the intensity-weighted distribution function obtained by CONTIN analysis of the correlation function embedded in Malvern software as well as electrophoretic mobility of the magnetic nanoparticles, which was converted to ζ-potential using the Smoluchowski equation.

A PerkinElmer 3110 atomic absorption spectrometer (AAS) was used to analyze the amount of iron in the particles by measuring the solution obtained after their mineralization with 68% HClO_4_/65% HNO_3_ (4/1 *v*/*v*) mixture at 80 °C for 20 min. The amount of phosphorus was determined after the mineralization and mixing with sulfuric acid, ammonium molybdate, ascorbic acid, and antimony potassium tartrate using a PerkinElmer Lambda 20 UV-Vis spectrometer at 690 nm.

ATR FTIR spectra were recorded on a PerkinElmer Paragon 1000PC spectrometer equipped with a Specac MKII Golden Gate single attenuated total reflection system with a diamond crystal; the angle of incidence was 45°.

Thermogravimetric analysis (TGA) was performed in air at 20–650 °C with a heating rate of 10 °C/min using a PerkinElmer TGA 7 analyzer and Pyris 1 software (Shelton, CT, USA).

Magnetic properties, namely coercivity and induced remanent magnetization were measured using an EV9 vibrating sample magnetometer (MicroSense; Lowell, MA, USA) at RT and maximum magnetic field of 1 T. Hysteresis loops were determined with different field steps from 500 Oe (0.05 T) in the highest field range (300 mT–1 T) to the finest step of 5 Oe (0.5 mT) in the lowest field range (−25–25 mT) at the intersections with magnetization and field axes. This allowed reliable determination of saturation remanent magnetization *M*_rs_, i.e., magnetization at zero field (the intersection with the magnetization axis), and coercive force *B*_c_ that is field necessary to induce zero magnetization (the intersection with the field axis). The two parameters were determined on both the ascending and descending branches of the hysteresis loop and resulting average values were considered. Saturation induced magnetization *M*_s_ was determined as the maximum induced magnetization after subtraction of the linear paramagnetic part of the loop at >0.5 T. Here, the ferrimagnetic response to external field was saturated and reflected by closed, reversible, and linear ascending and descending branches of the loop. Again, average *M*_s_ values were calculated, reflecting the concentration of ferrimagnetic substance in the specimen. To check its ability to acquire remanent magnetization, the measurements were performed in zero field after application of a step-wise increasing magnetic field, with 50 Oe (5 mT) field steps and 5000 Oe (0.5 T) maximum field. Remanent magnetization in true superparamagnetic particles at RT should decay rapidly to zero. Finally, after application of the maximum field, the same process was repeated, but with the field applied in the opposite direction. The field is necessary to remove *M*_rs_ named coercivity of remanence (*B*_cr_) and, similarly like *M*_rs_ and coercivity *B*_c_, it reflects magnetite particle size.

### 2.5. Antibacterial Activity of MNP@S-D and MNP@S-D-T

In 24-well plates (TPP; Trasadingen, Switzerland), overnight cultures of *S. aureus* and *E. coli* were resuspended in HBSS and diluted in LB (1 mL) to cultivate bacteria at 1 × 10^5^ colony-forming units (CFU) per mL. The MNP@S-D and MNP@S-D-T nanoparticles (5.5, 44, and 175 µg/mL) were added to the bacteria cultures and incubated at 37 °C with shaking (125 rpm). Culture without any treatment (NT) and containing ampicillin (150 µg/mL) served as a negative and positive control, respectively. After the exposure to nanoparticles, the bacteria aliquots were taken from the wells in time intervals of 0, 1, and 4 h, diluted 100×, 1000×, or 1,000,000×, seeded on LA plates, and incubated at 37 °C for 24 h. The procedure was repeated three times in duplicates and bacterial CFU/mL were calculated. The viability was calculated as a ratio of the number of living bacteria at given time interval to the number of living bacteria in NT. Data were analyzed by the GraphPad Prism5 software and two-way ANOVA followed by Bonferroni’s test.

## 3. Results and Discussion

### 3.1. Synthesis of MNP and Their Physicochemical Characterization

**Figure 1:**
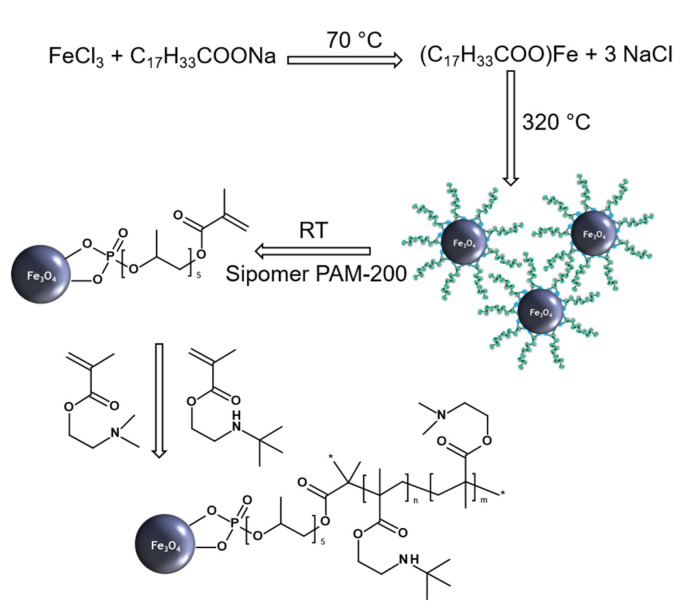
Synthesis of Fe_3_O_4_ nanoparticles, their modification with Sipomer PAM-200 and poly[2-(dimethylamino)ethyl methacrylate] or poly[2-(dimethylamino)ethyl methacrylate-*co*-2-(*tert*-butylamino)ethyl methacrylate].

OA-stabilized magnetite cores were synthesized by a thermal decomposition method in a non-polar high-boiling organic solvent (OD; Figure 1). The method allowed us to prepare magnetic nanoparticles with a very narrow size distribution, high crystallinity, and controlled diameter [39]. The synthesis proceeded in three steps: (i) formation of poly(iron oxo) clusters from an iron oleate serving as building blocks for the particle growth, (ii) short burst nucleation with formation of the particle seeds, and (iii) nanoparticle growth with increasing reaction temperature [36,40].

**Figure 2:**
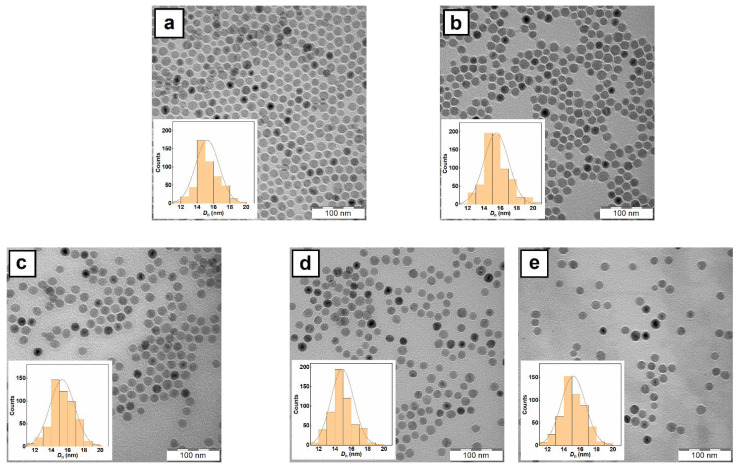
TEM micrographs of (**a**) MNP, (**b**) MNP@S, (**c**) MNP@S-D, (**d**) MNP@S-D-T1, and (**e**) MNP@S-D-T2.

**Figure 3:**
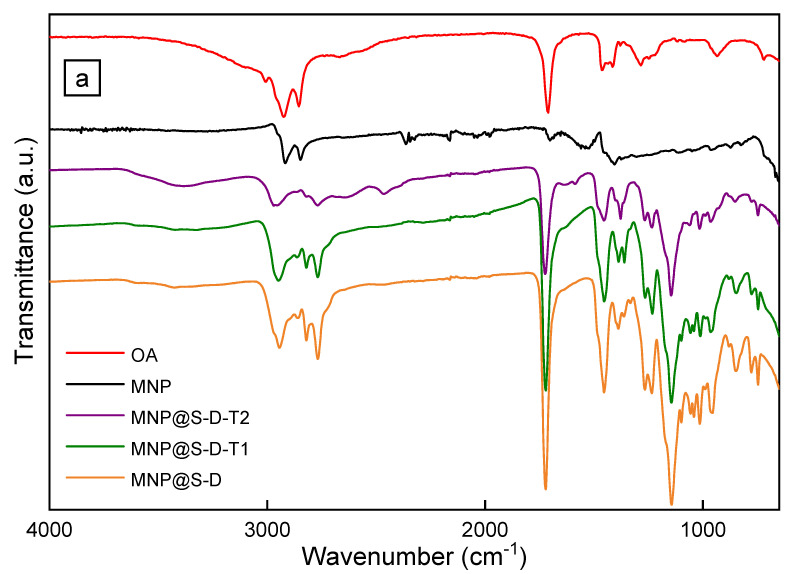

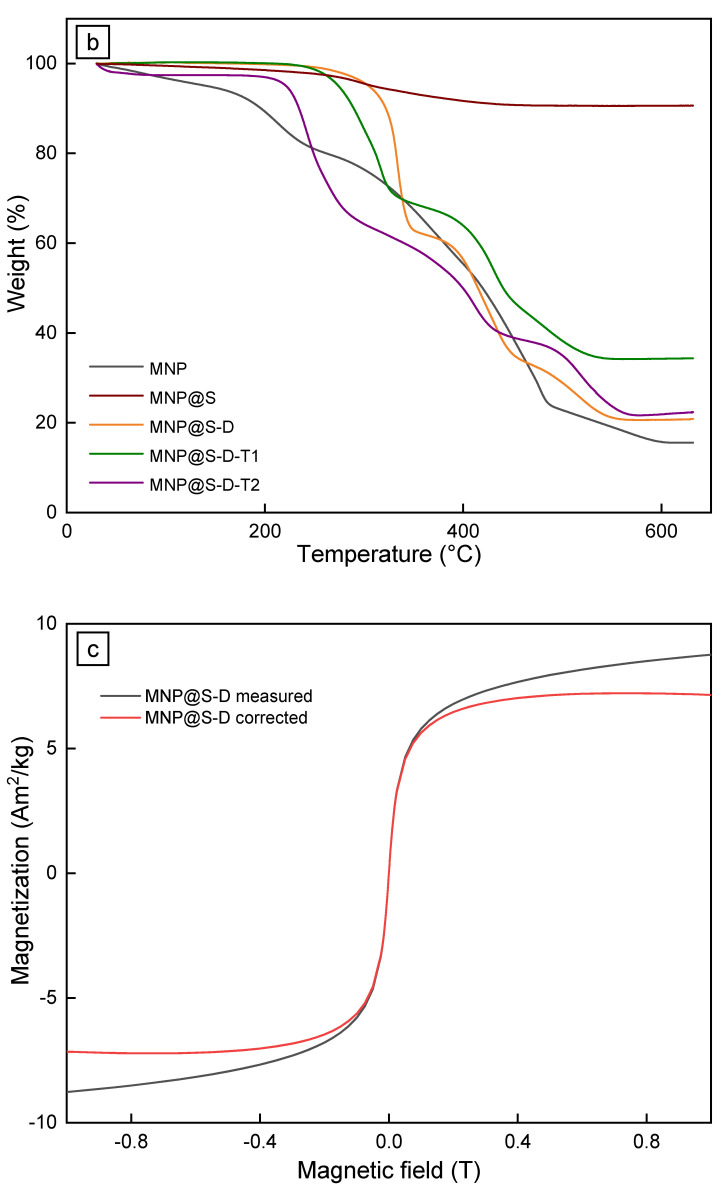
(**a**) FTIR and (**b**) TGA spectra of OA, MNP, MNP@S-D, MNP@S-D-T1, and MNP@S-D-T2. (**c**) Hysteresis loop of the MSP-S-D.

Due to the combination of short nucleation time and the presence of a stabilizer that prevents the nuclei aggregation and ensures the same conditions for each particle growth the resulting particles had controlled size (*D*_n_ = 16 nm) with low dispersity (*Ð* < 1.05) according to TEM (Figure 2a). Additionally, polydispersity (*PD* = 0.13) from DLS documented a very narrow particle size distribution (Table 1). The hydrodynamic diameter of nanoparticles measured in toluene was larger (*D*_h_ = 36 nm) than *D*_n_ due to the OA adsorbed on the particle surface. The FTIR spectra of MNP exhibited three peaks at 1460, 2844, and 2919 cm^−1^ attributed to ν_d_(CH) deformation vibration, and ν_s_(CH_2_) symmetric and ν_as_(CH_3_) asymmetric stretching vibration, respectively (Figure 3a). The bands at 1559 and 1703 cm^−1^ corresponded to ν_a_(COO^−^) asymmetric and ν(COOH) stretching vibrations, respectively. Therefore, FTIR spectroscopy confirmed the presence of OA coating on the Fe_3_O_4_ nanoparticle surface. According to TGA, the amount of organic compounds on the MNP surface equaled to 84 wt.%.

### 3.2. Surface Modification of MNP with Sipomer PAM-200

For the application of antibacterial MNP in disinfection of aqueous media, water-dispersible nanoparticles are required. However, the original magnetic particles contained hydrophobic OA coating and therefore they have to be modified with a hydrophilic polymer such as PDMAEMA to provide both dispersibility and colloidal stability of particles in water. To ensure efficient attachment of both PDMAEMA and its copolymers to the iron oxide surface, Sipomer PAM-200 terminated with phosphate groups was selected as a mediator. While the phosphate group enabled attachment of Sipomer PAM-200 to the iron oxide, which was superior to a mere physical adsorption, its reactive methacrylic group with a vinyl bond separated by a ten-carbon spacer from phosphate anchoring group allowed the free-radical copolymerization of DMAEMA monomer [16]. Analogously to OA, Sipomer PAM-200 due to its low contrast was not visible on the TEM micrograph (Figure 2b). The hydrodynamic diameter of MNP@S only slightly decreased to 28 nm compared to the initial nanoparticles, probably due to poorer solvation of Sipomer PAM-200 shell in toluene than that of OA (Table 1).

### 3.3. Modification of MNP@S with PDMAEMA and P(DMAEMA-TBAEMA)

**Figure 4:**
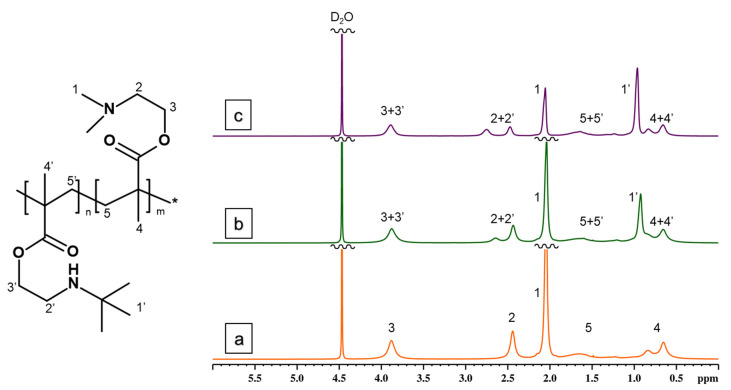
High-resolution ^1^H NMR spectra of (**a**) poly[2-(dimethylamino)ethyl methacrylate], (**b**) poly[2-(dimethylamino)ethyl methacrylate-*co-*2-(*tert*-butylamino)ethyl methacrylate] (0.75/0.25 mol/mol), and (**c**) poly[2-(dimethylamino)ethyl methacrylate-*co-*2-(*tert*-butylamino)ethyl methacrylate] (0.5/0.5 mol/mol) measured in D_2_O at 25 °C. Resonance assignment of PDMAEMA and PDMAEMA-TBAEMA copolymers is shown in the spectra.

From various antimicrobial polymers intended as a coating for the MNP, cationic PDMAEMA and PTBAEMA were selected due to their mucoadhesive, antibacterial, and stimuli-sensitive properties [25,41]. As the PTBAEMA has limited solubility in water, TBAEMA was copolymerized with a highly hydrophilic DMAEMA monomer to obtain water-dispersible antibacterial magnetic agents. At the beginning, different DMAEMA/TBAEMA molar ratios (1/0, 0.75/0.25, 0.5/0.5, 0.25/0.75) were studied in the preparation of polymers. While the MNP coated with copolymers prepared at the DMAEMA/TBAEMA ratio of 0.25/0.75 mol/mol were too hydrophobic and thus non-dispersible in water, the MNP@S-D, MNP@S-D-T1, and MNP@S-D-T2 prepared with DMAEMA/TBAEMA ratios equaling to 1/0, 0.75/0.25, and 0.5/0.5 mol/mol, respectively, were water-dispersible and therefore tested in further experiments (Table 1). The compositions of PDMAEMA and P(DMAEMA-TBAEMA) copolymers with different DMAEMA/TBAEMA molar ratios were investigated by ^1^H NMR spectroscopy by comparing methyl signals of side chains from DMAEMA and TBAEMA (Figure 4). The “1” resonance found at 2.1 ppm corresponded to (CH_3_)_2_ from DMAEMA chains, while signal “1” situated at ~1 ppm was related to (CH_3_)_3_ protons from TBAEMA. Calculated monomer ratios in the (co)polymers were in good agreement with those added in the polymeri-zation feed. The weight- (*M*_w_), number-average molar mass (*M*_n_), and polydispersity (*M*_w_/*M*_n_) of the polymers determined by SEC analysis showed that with increasing DMAEMA/TBAEMA ratio *M*_w_ of S-D, S-D-T1, and S-D-T2 polymers increased from 145 to 195 and 240 kDa, respectively; polydispersity remained rather low (*M*_w_/*M*_n_ = < 1.2) due to the purification of polymers by dialysis and/or chromatography.

In the next experiments, PDMAEMA- and P(DMAEMA-TBAEMA)-coated MNP were thoroughly physicochemically characterized. The TEM micrographs of MNP@S-D, MNP@S-D-T1, and MNP@S-D-T2 did not differ from those of starting Fe_3_O_4_ as the polymer coatings were not contrasted in the images (Figure 2c–e). The long-term colloidal stability of MNP@S-D, MNP@S-D-T1, or MNP@S-D-T2 in water (pH 6) was demonstrated by determination of *D*_h_ and ζ-potential, reaching 140, 110, or 98 nm and 48, 51, or 46 mV, respectively (Table 1). At this pH, amino groups of PDMAEMA were partially protonated and, as a result, the ξ-potential was quite high. It was typical that the hydrodynamic diameter *D*_h_ of particles measured by DLS in water was larger than the number-average diameter according to TEM due to several reasons. In DLS, the hydrodynamic diameter is determined by the autocorrelation function that compares the fluctuation of intensity of scattered light and provides z-average diameter (more sensitive to bigger particles). In contrast, TEM provides the number-average diameter that is in principle smaller than the *D*_h_. Moreover, the DLS measures the particles in water, where the polymer coatings can be swollen, while the TEM analyzes dry specimens. Finally, last but not least, the particles form doublets, triplets, and other small aggregates in water, whereas individual particles are calculated from TEM micrographs. The presence of phosphate groups on the particles was confirmed by the UV-Vis spectroscopy that revealed 0.88, 0.69, and 0.42 wt.% of phosphorus in the MNP@S-D, MNP@S-D-T1, and MNP@S-D-T2, respectively. These values agreed with the literature data, where Sipomer PAM-200-coated γ-Fe_2_O_3_ nanoparticles contained 1.49 wt.% of phosphorus [16]. The content of PDMAEMA-based coatings on the particles, *w*(polymer), was indirectly calculated not only from the amount of phosphorus, but also from the quantity of iron determined by AAS. Since the PDMAEMA or PTBAEMA did not contain any Fe or P, the content of PDMAEMA-based polymers on the particles could be calculated from the amount of the elements *w*(Fe) and *w*(P) according to Equation (1):

*w*(polymer) = 100 − w(Fe) × 100/72.4 − w(P) × 100/6.8 (1)

where 72.4 and 6.8 are percentages of Fe and P in neat Fe_3_O_4_ and Sipomer PAM-200, respectively. As a result, 67, 55, and 76 wt.% of methacrylate-based polymers was determined in the MNP@S-D, MNP@S-D-T1, and MNP@S-D-T2, respectively. It should also be noted that the MNP@S-D contained 14.4 wt.% Fe according to AAS, therefore, the calculated percentage of Fe_3_O_4_ and coating including Sipomer PAM-200 was 20 and 80 wt.%, respectively (Table 2). This was in agreement with the amount of coating determined by TGA (79 wt.%; Table 2). Similarly, MNP@S-D-T1 and MNP@S-D-T2 contained 65 and 82 wt.% of the P(DMAEMA-TBAEMA) copolymer according to AAS, while TGA revealed 66 and 78 wt.% of the polymer, which was again in a reasonable agreement.

The surface composition of MNP@S-D, MNP@S-D-T1, and MNP@S-D-T2 was further analyzed by FTIR spectroscopy (Figure 3a). The bands at 1148, 1454, and 1720 cm^−1^ were attributed to ν_as_(C-O-C) asymmetric stretching, δ(CH_2_) bending, and ν(C=O) stretching vibration, respectively. The two peaks located at 2861 and 2941 cm^−1^ were ascribed to ν_s_(CH_2_) symmetric and ν_as_(CH_3_) asymmetric stretching vibrations respectively. The broad band at 3380 cm^−1^ was assigned to ν(OH) stretching vibration of adsorbed water. The content of polymer on the particle surface was then analyzed by TGA (Figure 3b). In the MNP@S-D-T2, the initial weight loss observed at <100 °C was assigned to dehydration of particles. In the MNP@S-D, MNP@S-D-T1, and MNP@S-D-T2, the weight loss occurred at the temperatures ranges 219–584, 210–580, and 184–594 °C, respectively, that were associated with the decomposition of the polymer shell. After considering the Sipomer PAM-200 contribution (8 wt.%; Figure 3b), the content of PDMAEMA or P(DMAEMA-TBAEMA) was 77, 63, and 76 wt.% for MNP@S-D, MNP@S-D-T1, and MNP@S-D-T2, respectively. This amount of polymer coating was enough to ensure good colloidal stability of particles even after one month of storage. In addition, with increasing proportion of TBAEMA in the coating from 0 to 50 mol.%, the thermal stability of shell decreased, i.e., the decomposition of polymer started already from 219 to 184 °C, respectively. Consequently, it can be concluded that all three types of nanoparticles were stable at temperatures < 180 °C, which is quite sufficient in terms of prospective heat sterilization needed for biological experiments.

To prove the superparamagnetic character of the particles, their magnetic properties were examined. The measurements of remanent magnetization in zero field, i.e., acquisition of remanent magnetization as well as back-field remagnetization, yielded noisy curves close to zero. The inability of material to acquire stable remanent magnetization is typical for truly superparamagnetic particles, where the collective behavior due to interparticle interactions is absent. This was supported by the shape of hysteresis loops passing virtually through the origin and yielding coercive force *B*_c_ < 1 Oe (0.1 mT). Additional-ly, determination of *M*_rs_ from the induced magnetization measurements was obstructed by the very narrow loops (exemplified on Figure 3c; Table 1). Moreover, the data suggested that there were no interparticle magnetic interactions which might cause collective behavior and unwanted particle clustering in the magnetic field. Despite high degree of uncertainty, both parameters (*M*_rs_ and *B*_c_) suggested an increasing trend (Table 1). The only reliably determined magnetic parameter was saturation induced magnetization *M*_s_, reflecting the concentration of ferrimagnetic particles and confirming that the particles could be removed from aqueous suspensions using a magnet [42]. Stable single-domain and coarse multi-domain magnetite and maghemite have saturation magnetization of ~92 and ~80 A∙m^2^/kg, respectively; however, magnetic parameters of synthetic nanosized iron oxides are known to be size-dependent [43,44]. Coercive force and saturation remanence of our synthesized particles were well below the minimum values for nanosized magnetite, indicating that the particle size was ~10 nm [44]. Consequently, saturation magnetization of ~55 A∙m^2^/kg was used to estimate the upper limit of iron oxide concentration (Table 1). Hence, the content of Fe_3_O_4_ in MNP@S-D, MNP@S-D-T1, and MNP@S-D-T2 could be calculated, amounting to 13, 20, and 13 wt.%, respectively, that corresponded to 80 and 87 wt.% of coating (Table 2). These results approximately agreed with those from analysis of phosphorus and TGA.

### 3.4. Antibacterial Activity of the Particles

Cationic polymers, exemplified by thermo- and pH-responsive PDMAEMA, or hydrophobic TBAEMA are known to be promising antimicrobial agents [25,41]. Here, antimicrobial properties of MNP@S-D, MNP@S-D-T1, and MNP@S-D-T2 were investigated against two commonly used species of pathogen bacteria, namely, *S. aureus* and *E. coli*. These bacteria are Gram-negative (*E. coli*) and Gram-positive (*S. aureus*) containing structurally different cell walls. Biocidal processes involving the nanoparticle uptake by microorganisms consist of several steps, such as adsorption of the particles on bacterial cell surface, penetration through the cell wall, binding to cytoplasmic membrane and its disruption, release of cytoplasmic contents, and, finally, cell death [26,45]. The antibacterial mechanism of cationic DMAEMA and hydrophobic TBAEMA polymers might be based on a similar principle like the electrostatic interaction between a cationic compound and negatively charged constituents on the cell surface and/or hydrophobic interactions [25,26,34,46]; however, these effects can be strain-specific due to the distinct cell wall structures. In contrast to the Gram-positive bacteria, the Gram-negative ones possess an extra outer membrane composed of phospholipids, proteins, and lipopolysaccharides, which generally enable stronger protection against agents migrating to the cytoplasm [46]. In addition, Gram-negative bacteria require less charged and hydrophobic polymers to permeabilize the outer membrane [26].

**Figure 5:**
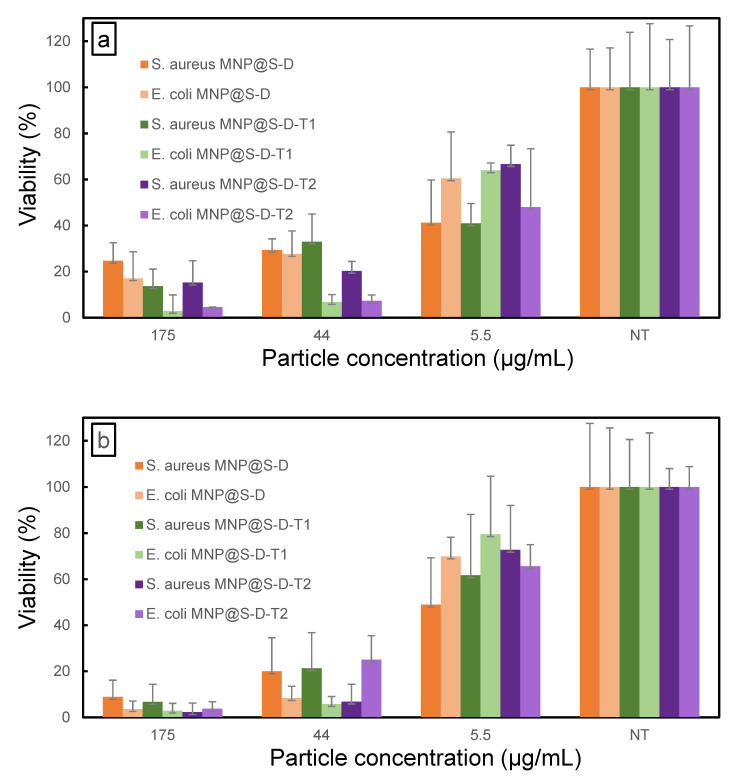

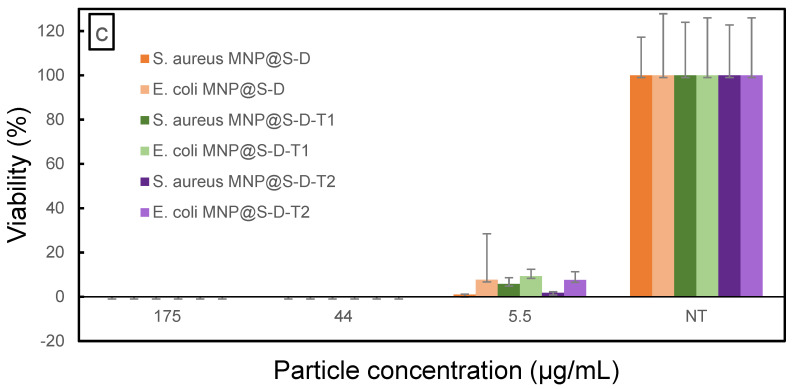
Viability of *S. aureus* and *E. coli* treated with MNP@S-D, MNP@S-D-T1, and MNP@S-D-T2 for (**a**) 0, (**b**) 1, and (**c**) 4 h. Culture without any treatment (NT) served as a negative control. Data are expressed as mean ± standard deviation of bacterial viability relative to control in three independent experiments. The results denoted significant differences at *p* < 0.001 (two-way ANOVA with Bonferroni’s test).

Antimicrobial activity of MNP@S-D, MNP@S-D-T1, and MNP@S-D-T2 (5.5, 44, and 175 µg/mL) against *S. aureus* and *E. coli* was determined after 0, 1, and 4 h of exposition. The effects were time- and concentration-dependent (Figure 5). The highest concentration of MNP@S-D-T1 and MNP@S-D-T2 (175 µg/mL) at time point 0 h decreased viability in both bacterial species more compared to that in the presence of MNP@S-D (Figure 5a). Higher concentrations of all types of nanoparticles (175 and 44 µg/mL) at time point 0 h resulted in lower viability of *E. coli* than that of *S. aureus*. A similar trend was observed after incubation of MNP@S-D and MNP@S-D-T1 for 1 h (Figure 5b). On the other hand, the lowest concentration of nanoparticles (5.5 µg/mL) incubated for 4 h induced higher viability of *E. coli* than that of *S. aureus* (Figure 5c). Moreover, the particles exhibited stronger antimicrobial activity after 1 h of incubation than at time point 0 h, indicating a beneficial effect of longer exposition of bacteria to nanoparticles in order to damage the cell wall. All types of particles (175 and 44 µg/mL) after 4 h of incubation affected bacterial viability of both cultures that decreased almost to zero (Figure 5c). Overall, the results showed that the MNP@S-D, MNP@S-D-T1, and MNP@S-D-T2 exhibited strong antimicrobial activity against both *E. coli* and *S. aureus* and their effectiveness depended on tested microorganism, concentration of particles, and their coating. In particular, MNP@S-D-T1 possessed superior biocidal properties.

## 4. Conclusions

Highly efficient antibacterial magnetic nanoparticles were prepared via a thermal decomposition that was followed by their modification with Sipomer PAM-200 and coating with PDMAEMA or P(DMAEMA-TBAEMA) obtained by radical (co)polymerization of 2-(dimethylamino)ethyl methacrylate and/or 2-tert-butylaminoethyl methacrylate. It is a great advantage of Sipomer PAM-200 that it consists of both phosphate group strongly interacting with iron oxide and methacrylate containing vinyl group prone to radical polymerization. The particles were uniform in size with diameters reaching 16 nm. Magnetic properties of the cationic polymer-coated nanoparticles clearly reflected their superparamagnetic character, without signs of clustering. As a result, the particles were easily attracted by even low external magnetic field. The antimicrobial effects of MNP@S-D, MNP@S-D-T1, and MNP@S-D-T2 against two different kinds of bacteria, *S. aureus* and *E. coli*, were found to be concentration- and time-dependent. Antibacterial properties were enhanced with increasing concentration of the nanoparticles and time of incubation. In particular, the MNP@S-D-T1 even at low concentrations (44 µg/mL) exhibited a strong antibacterial effect on both microbial cultures. This makes these particles suitable as an efficient and reusable antibacterial agent.

## Figures and Tables

**Table 1:** Physicochemical characterization of the iron oxide nanoparticles.

Particles	*D*_n_ (nm)	*Ð*	*D*_h_ (nm)	*PD*	ζ-Potential (mV)	*B*_c_ (mT)	*M*_rs_ (10^−3^ A∙m^2^/kg)	*M*_s_ (A∙m^2^/kg)
MNP	16	1.02	36 ^a^	0.13	-	-	-	-
MNP@-S	16	1.03	28 ^a^	0.16	-	-	-	-
MNP@S-D	16	1.03	140 ^b^	0.19	48	0.0292	6.584	7.190
MNP@S-D-T1	16	1.02	110 ^b^	0.18	51	0.0371	8.851	11.228
MNP@S-D-T2	16	1.03	98 ^b^	0.17	46	0.0469	10.395	7.163

*D*_n_—number-average particle diameter (TEM); *Ð*—dispersity (TEM); *D*_h_—hydrodynamic diameter (DLS; ^a^ in toluene or ^b^ in water); *PD*—polydispersity index (DLS); *B*_c_—coercive force; *M*_rs_—saturation remanent magnetization; *M*_s_—saturation magnetization.

**Table 2:** Results of UV-Vis analysis and AAS of MNP modified with poly[2-(dimethylamino)ethyl methacrylate] and poly[2-(dimethylamino)ethyl methacrylate-*co*-2-*tert*-butylaminoethyl methacrylate].

Particles	Content (wt.%)		Coating ^c^ (wt.%)		
	P ^a^	Fe ^b^	UV-Vis	Magnetometry	TGA
MNP@S-D	0.88	14.4	80	87	79
MNP@S-D-T1	0.69	25.0	65	80	66
MNP@S-D-T2	0.42	13.2	82	87	78

^a^ UV-Vis analysis; ^b^ AAS; ^c^ including Sipomer PAM-200.

## Data Availability

All data are contained within the article.

## Abbreviations

AAS	atomic absorption spectroscopy
*B*_c_	coercive force
CFU	colony-forming units
*Ð*	dispersity
*D*_h_	hydrodynamic diameter
DLS	dynamic light scattering
*D*_n_	number-average diameter
*D*_w_	weight-average diameter
HBSS	Hank’s balanced salt solution
LA	Luria agar
LB	Luria broth
MNP	magnetic nanoparticles
MNP@S	MNP coated by Sipomer PAM-200
MNP@S-D	MNP coated by Sipomer PAM-200 and modified with poly[2-(dimethylamino)ethyl methacrylate]
MNP@S-D-T1	MNP coated by Sipomer PAM-200 and modified with 2-(dimethylamino)ethyl methacrylate and 2-*tert*-butylaminoethyl methacrylate copolymer (0.75:0.25 mol/mol)
MNP@S-D-T2	MNP coated by Sipomer PAM-200 and modified with 2-(dimethylamino)ethyl methacrylate and 2-*tert*-butylaminoethyl methacrylate copolymer (0.5:0.5 mol/mol)
*M*_s_	saturation magnetization
*M*_rs_	remanent saturation magnetization
OA	oleic acid
OD	octadec-1-ene
*PD*	polydispersity
PDMAEMA	poly[2-(dimethylamino)ethyl methacrylate]
PTBAEMA	poly(2-*tert*-butylaminoethyl methacrylate)
TEM	transmission electron microscope

## References

[1] Aslam B., Wang W., Arshad M.I., Khurshid M., Muzammil S., Rasool M.H., Nisar M.A., Alvi R.F., Aslam M.A., Qamar M.U. (2018). Antibiotic resistance: A rundown of a global crisis. Infect. Drug Resist..

[2] Fair R.J., Tor Y. (2014). Antibiotics and bacterial resistance in the 21st century, perspectives in medicinal chemistry. Perspect. Medicin. Chem..

[3] Hoffman S.J., Caleo G.M., Daulaire N., Elbe S., Matsoso P., Mossialos E., Rizvig Z., Røttingenh J.A. (2015). Strategies for achieving global collective action on antimicrobial resistence. Bull. World Health Organ..

[4] Guo N., Cang F., Wang Z., Zhao T., Song X., Farris S., Li Y., Fu Y. (2021). Magnetism and NIR dual-response polypyrrole-coated Fe_3_O_4_ nanoparticles for bacteria removal and inactivation. Mater. Sci. Eng. C Mater. Biol. Appl..

[5] Fatima F., Siddiqui S., Khan W.A. (2021). Nanoparticles as novel emerging therapeutic antibacterial agents in the antibiotics resistant era. Biol. Trace Elem. Res..

[6] Raza S., Matuła K., Karoń S., Paczesny J. (2021). Resistance and adaptation of bacteria to non-antibiotic antibacterial agents: Physical stressors, nanoparticles, and bacteriophages. Antibiotics.

[7] Wang L., Hu C., Shao L. (2017). The antimicrobial activity of nanoparticles: Present situation and prospects for the future. Int. J. Nanomed..

[8] De M., Ghosh P.S., Rotello V.M. (2008). Applications of nanoparticles in biology. Adv. Mater..

[9] Gupta A.K., Naregalkar R.R., Vaidya V.D., Gupta M. (2007). Recent advances on surface engineering of magnetic iron oxide nanoparticles and their biomedical applications. Nanomedicine.

[10] Kostiv U., Farka Z., Mickert M.J., Gorris H.H., Velychkivska N., Pop-Georgievski O., Pastucha M., Odstrčilíková E., Skládal P., Horák D. (2020). Versatile bioconjugation strategies of PEG-modified upconversion nanoparticles for bioanalytical applications. Biomacromolecules.

[11] Santos S., Ramalho P., Viana A.T., Lopes A.R., Gonçalves A.G., Nunes O.C., Pereira F.R., Soares S. (2021). Feasibility of using magnetic nanoparticles in water disinfection. J. Environ. Manag..

[12] Armijo L.M., Jain P., Malagodi A., Fornelli Z., Hayat A., Rivera A.C., French M., Smyth H., Osiński M. (2015). Inhibition of bacterial growth by iron oxide nanoparticles with and without attached drug: Have we conquered the antibiotic resistance problem?. Proc. SPIE.

[13] De Toledo L.A.S., Rosseto H.C., Bruschi M.L. (2017). Iron oxide magnetic nanoparticles as antimicrobials for therapeutics. Pharm. Dev. Technol..

[14] Portet D., Denizot B., Rump E., Lejeune J.-J., Jallet P. (2001). Nonpolymeric coatings of iron oxide colloids for biological use as magnetic resonance imaging contrast agents. J. Colloid Interface Sci..

[15] Konefał M., Cérnoch P., Patsula V., Pavlova E., Dybal J., Załęski K., Zhigunov A. (2021). Enhanced ordering of block copolymer thin films upon addition of magnetic nanoparticles. ACS Appl. Mater. Interfaces.

[16] Tocchio A., Horák D., Babič M., Trchová M., Veverka M., Beneš M.J., Šlouf M., Fojtík A. (2009). Magnetic poly(glycidyl metha-crylate) particles prepared in the presence of surface-modified γ-Fe_2_O_3_. J. Polym. Sci. A.

[17] Jain A., Duvvuri L.S., Farah S., Beyth N., Domb A.J., Khan W. (2014). Antimicrobial polymers. Adv. Healthc. Mater..

[18] Milovic N.M., Wang J., Lewis K., Klibanov A.M. (2005). Immobilized *N*-alkylated polyethylenimine avidly kills bacteria by rupturing cell membranes with no resistance developed. Biotechnol. Bioeng..

[19] Timofeeva L., Kleshcheva N. (2011). Antimicrobial polymers: Mechanism of action, factors of activity, and applications. Appl. Microbiol. Biotechnol..

[20] Cerichelli G., La Mesa C., Luchetti L., Mancini G. (2000). Role of counterions in the catalytic activity and phase equilibria of phosphonium salts in water. Langmuir.

[21] Hugues C., Bessy C., Bartolomeo P., Margaillan A. (2003). Complexation of an acrylic resin by tertiary amines: Synthesis and chara-cterisation of new binders for antifouling paints. Eur. Polym. J..

[22] Kanazawa A., Ikeda T. (2000). Multifunctional tetracoordinate phosphorus species with high self-organizing ability. Coord. Chem. Rev..

[23] Li G., Shen J., Zhu Y. (2000). A study of pyridinium-type functional polymers. III. Preparation and characterization of insoluble pyridinium-type polymers. J. Appl. Polym. Sci..

[24] Nurdin N., Helary G., Sauvet G. (1993). Biocidal polymers active by contact. III. Ageing of biocidal polyurethane coatings in water. J. Appl. Polym. Sci..

[25] Rawlinson L.B., Ryan S.M., Mantovani G., Syrett J.A., Haddleton D.M., Brayden D.J. (2010). Antibacterial effects of poly(2-(dimethylamino ethyl)methacrylate) against selected Gram-positive and Gram-negative bacteria. Biomacromolecules.

[26] Ward M., Sanchez M., Elasri M.O., Lowe A.B. (2006). Antimicrobial activity of statistical polymethacrylic sulfopropylbetaines against Gram-positive and Gram-negative bacteria. J. Appl. Polym. Sci..

[27] Yancheva E., Paneva D., Maximova V., Mespouille L., Dubois P., Manolova N., Rashkov I. (2007). Polyelectrolyte complexes between (cross-linked) *N*-carboxyethylchitosan and (quaternized) poly[2-(dimethylamino)ethyl methacrylate]:  Preparation, cha-racterization, and antibacterial properties. Biomacromolecules.

[28] Gu Z., Yuan Y., He J., Zhang M., Ni P. (2009). Facile approach for DNA encapsulation in functional polyion complex for triggered intracellular gene delivery: Design, synthesis, and mechanism. Langmuir.

[29] Keely S., Ryan S.M., Haddleton D.M., Limer A., Mantovani G., Murphy E.P., Colgan S.P., Brayden D.J. (2009). Dexamethasone–pDMAEMA polymeric conjugates reduce inflammatory biomarkers in human intestinal epithelial monolayers. J. Control. Release.

[30] Zhu S., Yang N., Zhang D. (2009). Poly(*N,N*-dimethylaminoethyl methacrylate) modification of activated carbon for copper ions removal. Mater. Chem. Phys..

[31] Kusumo A., Bombalski L., Lin Q., Matyjaszewski K., Schneider J.W., Tilton R.D. (2007). High capacity, charge-selective protein uptake by polyelectrolyte brushes. Langmuir.

[32] Geng Q.R., Xiao J.G., Yang B., Wang T., Du J.Z. (2015). Rationally engineering dual missions in one statistical copolymer nanocapsule: Bacterial inhibition and polycyclic aromatic hydrocarbon capturing. ACS Macro Lett..

[33] Lenoir S., Pagnoulle C., Galleni M., Compere P., Jerome R., Detrembleur C. (2006). Polyolefin matrixes with permanent antibacterial activity: Preparation, antibacterial activity, and action mode of the active species. Biomacromolecules.

[34] Zuo H., Wu D., Fu R. (2012). Preparation of antibacterial poly(methyl methacrylate) by solution blending with water-insoluble antibacterial agent poly[(*tert*-buty1amino) ethyl methacrylate]. J. Appl. Polym. Sci..

[35] Huang C.-L., Lee K.-M., Liu Z.-X., Lai R.-Y., Chen C.-K., Chen W.-C., Hsu J.-F. (2020). Antimicrobial activity of electrospun polyvinyl alcohol nanofibers filled with poly[2-(*tert*-butylaminoethyl) methacrylate]-grafted graphene oxide nanosheets. Polymers.

[36] Patsula V., Petrovský E., Kovářová J., Konefal R., Horák D. (2014). Monodisperse superparamagnetic nanoparticles by thermolysis of Fe(III) oleate and mandelate complexes. Colloid Polym. Sci..

[37] Patsula V., Horák D., Kučka J., Macková H., Lobaz V., Francová P., Herynek V., Heizer T., Páral P., Šefc L. (2019). Synthesis and modification of uniform PEG-neridronate-modified magnetic nanoparticles determines prolonged blood circulation and biodistribution in a mouse preclinical model. Sci Rep..

[38] Shatan A.B., Venclíková K., Zasońska B.A., Patsula V., Pop-Georgievski O., Petrovský E., Horák D. (2019). Antibacterial silver-conjugated magnetic nanoparticles: Design, synthesis and bactericidal effect. Pharm. Res..

[39] Patsula V., Moskvin M., Dutz S., Horák D. (2016). Size-dependent magnetic properties of iron oxide nanoparticles. J. Phys. Chem. Solids..

[40] Patsula V., Kosinová L., Lovrić M., Ferhatovic Hamzić L., Rabyk M., Konefal R., Paruzel A., Šlouf M., Herynek V., Gajović S. (2016). Superparamagnetic Fe_3_O_4_ nanoparticles: Synthesis by thermal decomposition of iron(III) glucuronate and application in magnetic resonance imaging. ACS Appl. Mater. Interfaces.

[41] Thomassin J.-M., Lenoir S., Riga J., Jérôme J., Detrembleur C. (2007). Grafting of poly[2-(*tert*-butylamino)ethyl methacrylate] onto polypropylene by reactive blending and antibacterial activity of the copolymer. Biomacromolecules.

[42] Horák D., Sharma S.K., Javed Y. (2020). Magnetic nano and microparticles in life sciences and medical imaging. Magnetic Nanoheterostructures: Diagnostic, Imaging and Treatment.

[43] Lee J.S., Cha J.M., Yoon J.Y., Lee J.-K., Kim Y.K. (2015). Magnetic multi-granule nanoclusters: A model system that exhibits universal size effect of magnetic coercivity. Sci. Rep..

[44] Li Q., Kartikowati C.W., Horie S., Ogi T., Iwaki T., Okuyama K. (2017). Correlation between particle size/domain structure and magnetic properties of highly crystalline Fe_3_O_4_ nanoparticles. Sci. Rep..

[45] Chen C.-K., Lee M.-C., Lin Z.-I., Lee C.-A., Tung Y.-C., Lou C.-W., Law W.-C., Chen N.-T., Lin K.-Y.A., Lin J.H. (2019). Intensifying the antimicrobial activity of poly[2-(*tert*-butylamino)ethyl methacrylate]/polylactide composites by tailoring their chemical and physical structures. Mol. Pharm..

[46] Fu Y., Wang Y., Huang L., Xiao S., Chen F., Fan P., Yang J. (2018). Salt-responsive “killing and release” antibacterial surfaces of mixed polymer brushes. Ind. Eng. Chem. Res..

